# Polysplenia syndrome in adulthood: a case report

**DOI:** 10.11604/pamj.2022.41.67.29014

**Published:** 2022-01-25

**Authors:** Manal Cherkaoui Malki, Mustapha Outznit, Salma Mechhor, Boutaina Bouibaouen, Lambert Nkurunziza, Hicham El Bacha, Nadia Benzzoubeir, Fatime Zahrae Laamrani, Laila Jroundi, Ikram Errabih

**Affiliations:** 1 Department of Gastroentero-Hepatology and Proctology “Médecine B” department, University Hospital Ibn Sina, Rabat, Morocco,; 2 Emergency Radiology Department, University Hospital Ibn Sina, Rabat, Morocco

**Keywords:** Polysplenia, adult, incidental, malformations, case report

## Abstract

Polysplenia syndrome mainly described in pediatrics; rarely and incidentally in adulthood. Most patients had their diagnosis done during childhood due to the frequent association to cardiac anomalies that speak for themselves earlier in life. Multiple spleens, cardiac defect and vascular malformation of the inferior vena cava with azygos or hemiazygos continuation are the most frequent observed malformations. Our patient was one this rarest adulthood incidental diagnosis, who presented in the emergency department for nephritic colic, and while imaging for this, multiples spleens and other visceral malformations were diagnosed. Hopefully, cardiac ultrasound hadn't showed any cardiac malformation and the patient was discharged aware of this condition. Through this publication we report the possible incidental diagnosis of polysplenia condition and highlight the fact that people with such important malformation can lead a normal life, and only awareness should be given for future surgeries, instrumental treatment or else.

## Introduction

Polysplenia syndrome (PS) is a rare condition defined as the presence of two or more spleens associated to various thoracic or abdominal abnormalities, thus defining situs ambiguous. In literature, situs ambiguous or polysplenia syndrome is mainly described in pediatrics and rarely in adults [1]. This can be explained by the frequent association to congenital heart disease that prevent children from reaching adult age [2]. Other common malformations are described such as vascular, pancreatic, duodenal and abnormal lung lobe number. In adults, PS is usually diagnosed incidentally on medical imaging exams while looking for other causes. Its management and prognosis depend on the type of the malformation. Generally, in adult, no specific treatment is preconized, only awareness for later potential surgeries or other treatments is given. Through this paper, we report a rare case of an adult with polysplenia syndrome with vascular and pancreatic abnormalities detected during Computed tomography (CT) scan imaging for nephritic colic.

## Patient and observation

**Patient information:** a 60-year-old man with no personal medical or surgical history presented to the emergency department for acute left renal colic. No particular family history of malformation or handicap was noted.

**Clinical findings:** general examination found a patient with tachycardia of 105 beats per minute, apyretic, with no jaundice, abdominal examination found lower left back pain with no signs of spleen or liver disease. His abdomen was non-tender with no distension. There were no palpable masses or organomegaly.

**Timeline of current episode:** January 2019: acute left renal colic, CT-scan performed the same day, showed ureteral calculi plus multiples spleens, visceral and vascular anomalies. Patient was discharged the same day.

**Figure 1:**
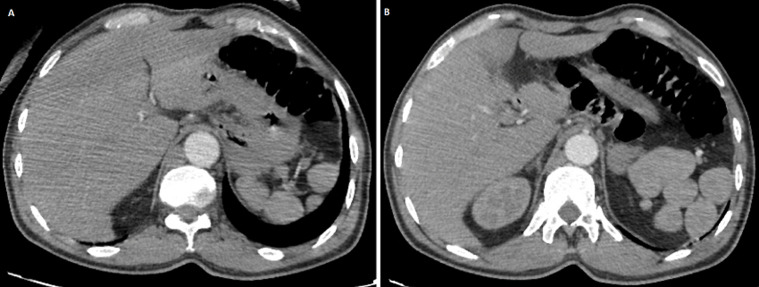
(A, B) multiple rounded soft tissue density structures at the anatomical site of spleen corresponding to multiple spenules

**Figure 2:**
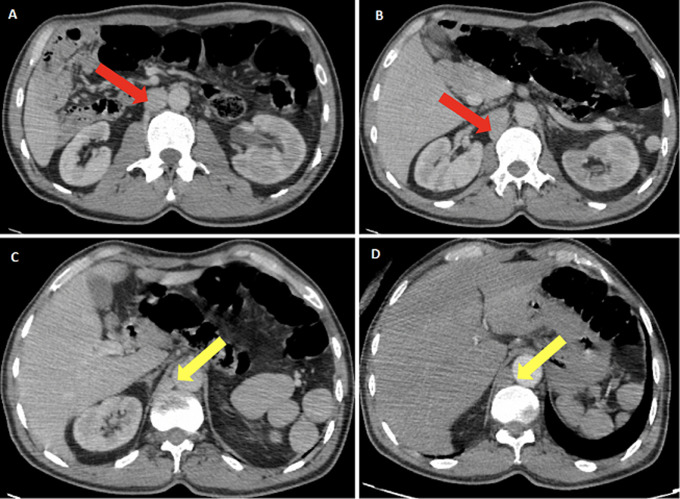
(A, B, C, D) absence of the hepatic segment of the inferior vena cava (red arrow) with azygos continuation (yellow arrow)

**Figure 3:**
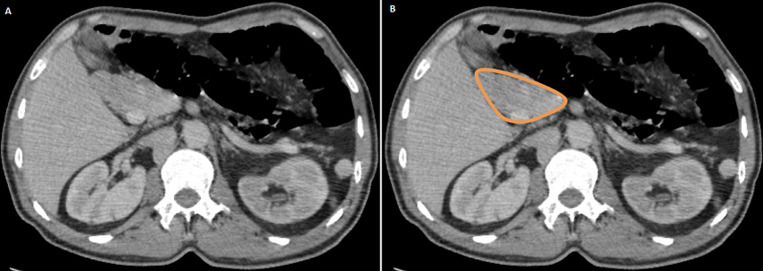
(A, B) short and rounded pancreatic head adjacent to the duodenum with the absence of tail and part of body

**Diagnostic assessment:** urinary tract CT-scan performed, found in addition to left ureteral calculi, multiple abdominal abnormalities, associating, multiple spenules on the left side of the upper abdomen along the greater curvature of the stomach (Figure 1), interruption of the inferior vena cava with azygos vein continuity (Figure 2), partial agenesis of dorsal pancreas (Figure 3), with no suprarenal portion, and hepatic veins draining directly into the right atrium. There was no abnormal bowel rotation; the liver was right-sided with no gall bladder or portal vein abnormality. Cardiac ultrasound performed hadn't shown any cardiac defect, and no renal or ureteral malformation were noted. Laboratory test performed, has shown no biological signs of hypersplenism. Platelets and white blood cells were normal, the complete blood count was normal, C-reactive protein test (CRP) was less than 10 mg/L and liver function tests were no normal. Since anomalies have no consequences, the prognosis is good.

**Diagnosis:** according to this, the diagnosis of left ureteral calculi with the incidental diagnosis of polysplenia syndrome was retained.

**Therapeutic interventions:** for ureteral calculi, 100 mg parenteral intravenous infusion of nonsteroidal anti-inflammatory was administrated. Behavior modification and preventative management were advised waiting for his shock wave lithotripsy. No monitoring for PS was preconized but awareness of his condition was given.

**Follow-up and outcome of interventions:** pain disappears few minutes after drugs administration. Patient was discharged with a 7 days prescription of oral nonsteroidal anti-inflammatory. No specific monitoring or follow-up was set for his PS.

**Patient perspective:** patient was astonished when he heard about his condition.

**Informed consent:** patient gave informed consent.

## Discussion

Polysplenia syndrome is a rare condition with an incidence of 1 per 250,000 live births [1], usually associated to severe cardiovascular abnormalities with a reported mortality of 75% by the age of five [2]. However, 5 to 10% of PS patients are asymptomatic with no severe cardiac anomalies explaining the late incidental diagnosis in adulthood [2]. A confusing list of terms is used to refer to PS; heterotaxy, situs ambigus, or left isomerism, though all are synonyms. Situs ambigus or heterotaxy is defined as the malposition of the thoracic and abdominal organs associated or not to vascular abnormalities. It is subclassified as situs ambiguous with polysplenia and situs ambiguous with asplenia.

Polysplenia syndrome is a bilateral left-sidedness, with bilateral bilobed lungs, bilateral pulmonary atria, and abnormal location of abdominal organs with multiple spleens; although there is no characteristic pathognomonic abnormality, one of the most common is the azygos or hemiazygos continuation of the inferior vena cava. As described in literature, our patient has indeed azygos continuation of inferior vena cava with multiple spleens. It´s important to point out, although it's very rare, the cases presenting only one spleen and common characteristics of heterotaxy, that is classified as polysplenia syndrome as well [3,4]. It affects more females than males [4,5], its exact cause is still unknown, but factors causing PS are thought to be an association of embryonic and genetic components [6] explained by the disruption of left-right axis determination during early embryonic development and by the mutations in some of the 80 genes required for normal asymmetric left-right organ development [7,8]. Since there is no pathognomonic anomaly characterizing PS, it is suggested that all the association of anomalies should be described individually. Cardiac anomalies are less common in polysplenia than asplenia but concerns nearly 50%-90% of cases, thus, only 10% are expected to reach adulthood [9].

Many types of cardiac defects are reported in literature, we mention, bilateral pulmonary atria opening with right pulmonary veins into the right atrium, and anomaly of the apex pointing. In a case series, Peoples *et al*. found that bilateral bilobed lungs were present in 48.9% of autopsies done to patients with polysplenia syndrome [10]. Our patient had normal lung segmentation, but had several abdominal abnormalities, associating, venous anomaly with the interruption of the inferior vena cava with azygos continuation, that is considered as the second most common abnormality associated to PS [11], and partial agenesis of dorsal pancreas which its association to PS is frequent because both organs develop in the dorsal mesogastrium [12]. Other pancreatic anomalies have been described: short pancreas, annular pancreas, pancreas divisum and pancreas malrotation. Intestinal malrotation can be seen in 60.4% cases of polysplenia. A midline liver, a polylobulated gallbladder, biliary atresia and a preduodenal portal vein can also be seen. In our patient we didn´t find any of these abnormalities.

The management of PS depends on the age, surgery is suggested to treat cardiac defects in childhood which prognosis is usually poor. Symptomatic management should be taken in adulthood, however, special attention should be made in case of preduodenal portal vein, intestinal malrotation or vascular anomalies so it can avoid complications during surgical procedures, there´s no recommendation about the follow-up and surveillance. Our patient, after a full checkup, had no anomaly requiring surgery or special treatment for polysplenia syndrome. He had been discharged after ureterolithiasis treatment. The only limitation of this present case, is that we don't have any long-term follow-up, but its rarest description in literature makes this case report worth reading.

## Conclusion

Polysplenia syndrome is a rare condition involving multiple congenital malformations which diagnosis in adulthood is usually incidental while imaging for other causes. Unlike in childhood, polysplenia syndrome in adulthood doesn´t require any specific treatment, only patient´s and doctor's awareness.
